# Innovative platforms for data aggregation, linkage and analysis in the context of pandemic and epidemic intelligence

**DOI:** 10.2807/1560-7917.ES.2023.28.24.2200860

**Published:** 2023-06-15

**Authors:** Beth Blauer, John S Brownstein, Lauren Gardner, Moritz UG Kraemer, Zoila Beatriz Leiva Rioja, Edouard Mathieu, Isabel Redies, Oliver W Morgan

**Affiliations:** 1 Johns Hopkins School of Public Health, Baltimore, Maryland, United States; 2 Boston Children’s Hospital, Boston, Massachusetts, United States; 3 Johns Hopkins Whiting School of Engineering, Baltimore, Maryland, United States; 4 University of Oxford, Oxford, United Kingdom; 5 Pandemic Sciences Institute, University of Oxford, United Kingdom; 6 CPC Analytics, Berlin, Germany; 7 Our World in Data, Oxford, United Kingdom; 8 World Health Organization Hub for Pandemic and Epidemic Intelligence, Berlin, Germany

**Keywords:** outbreak, data analysis, epidemics, pandemics, public health intelligence

## Abstract

During the COVID-19 pandemic, open-access platforms that aggregate, link and analyse data were transformative for global public health surveillance. This perspective explores the work of three of these platforms: Our World In Data (OWID), Johns Hopkins University (JHU) COVID-19 Dashboard (later complemented by the Coronavirus Resource Center), and Global.Health, which were presented in the second World Health Organization (WHO) Pandemic and Epidemic Intelligence Innovation Forum. These platforms, operating mostly within academic institutions, added value to public health data that are collected by government agencies by providing additional real-time public health intelligence about the spread of the virus and the evolution of the public health emergency. Information from these platforms was used by health professionals, political decision-makers and members of the public alike. Further engagement between government and non-governmental surveillance efforts can accelerate the improvements needed in public health surveillance overall. Increasing the diversity of public health surveillance initiatives beyond the government sector comes with several benefits: technology innovation in data science, engagement of additional highly skilled professionals, greater transparency and accountability for government agencies, and new opportunities to engage with members of society.

## Introduction

Since the start of the COVID-19 pandemic in 2020, platforms that aggregate, link and analyse COVID-19 data have provided open access to real-time public health intelligence information for health professionals, political decision-makers and members of the public. These aggregation platforms, operating mostly within academic institutions, have successfully added value to public health data that are collected primarily by government agencies. They also provided high quality visualisations, analytics and insights about the pandemic. Some of the platforms grew in popularity through their use by global news media to such an extent that they became household names.

During the second World Health Organization (WHO) Pandemic and Epidemic Intelligence Innovation Forum, which convened on 12 May 2022, three highly dynamic aggregation platforms reporting on COVID-19 were presented: Our World In Data (OWID) [1], Johns Hopkins University (JHU) COVID-19 Dashboard [2,3], which was later complemented by the setup of the Coronavirus Resource Center [4], and Global.Health [5]. The meeting provided an opportunity to discuss topics such as: data quality challenges for comprehensive data curation and validation, providing transparency and building trust by implementing open-access approaches, protecting data with innovative technologies, enhancing user’s comprehension of the data, and incentivising local and national governments to share data. 

This perspective piece draws on the content of the meeting by organically bringing together key arguments to improve public health practice for future pandemics and epidemics. Although these are not the only available platforms offering a similar output, they are good examples of innovative bottom-up approach initiatives outside the public sector. All three platforms originated in academia very early in the pandemic. They started very small and with very limited budgets, yet managed to become reliable sources for decision-makers and the public. In addition, they were successful in communicating science to the public, thanks to the interdisciplinarity in their teams. The platform teams established strong ties with collaborators from around the world, based on a common interest to provide better data for decision-making. The aim of this perspective is to summarise the work of three platforms that provided crucial inputs for public health decisions during the COVID-19 pandemic and to offer a contribution for the discussion of data aggregation for pandemic and epidemic intelligence.

## Initiating collaborative, bottom-up, interdisciplinary efforts to aggregate and link data

Upon the emergence of the severe acute respiratory syndrome coronavirus 2 (SARS-CoV-2) in early 2020, different communities of practice were seeking trusted, open-access information platforms that showed the evolution of the global epidemiological situation. Several initiatives responded by creating platforms to aggregate, link and analyse data that were being generated by countries and healthcare systems around the world. Three innovating examples, based on their intrinsic nature, are OWID, JHU and Global.Health.

Our World In Data, a platform based at the University of Oxford, in the United Kingdom, compiled a large dataset with a variety of metrics: confirmed cases, deaths, intensive care unit (ICU) admissions, epidemic reproduction rates and vaccinations, among others. Sources used were national public health agencies, WHO COVID-19 data, the European Centre for Disease Prevention and Control (ECDC), the World Bank, the Organisation for Economic Co-operation and Development, and JHU Center for Systems Science and Engineering (CSSE) among others [6]. Users could interact with these metrics through the OWID COVID-19 data explorer. The CSSE at JHU created what is possibly the most well-known COVID-19 Dashboard, which provided public access to daily updates of cases and deaths, as well as vaccine doses by country [7]. Up to 10 March 2023, JHU had aggregated data on 676,609,955 cases and 6,881,955 deaths worldwide from hundreds of sources, including local, state and national governmental health authorities and other validated data aggregators [8]. The work of both platforms was also closely linked; thus, OWID used data on cases and deaths aggregated by JHU as an input for their visualizations, and when JHU started collecting vaccination data, they compared them with the data aggregated by OWID on a permanent basis. Global.Health took a novel and complimentary approach by creating a standardised line list of individual-level data based on open sources and voluntary submissions from countries. This line-listed dataset contains over 100 million anonymised cases from over 100 countries, including where available data such as demographics, symptoms, pre-existing conditions and travel history, among other details. The recording and sharing of these types of data are crucial to understanding the transmissibility, routes of transmission and risk factors for infection, as well as to inform planning of response and containment efforts [9].

## Ensuring data quality through comprehensive data curation and validation

All three initiatives faced many challenges when setting up their data repositories. Initially, aggregated data were largely scraped manually from government websites, social media and news media outlets [10]. As the pandemic evolved, these platforms developed increasing levels of automation, building highly customised scrapers to gather data from a wide variety of sources. Global.Health additionally sought data from peer-reviewed scientific journal articles [11].

For all three platforms, the processes and infrastructure required for the curation of data were largely developed from scratch. Even with the enhancement of automation, the human factor remained important: curation teams with data analytics and public health skills have been key to ensuring the information in their repositories meets high-quality standards. They continuously checked its data against other sources to identify errors or misalignments, ensure data accuracy, and understand and address errors.

While robust data-checking routines are important for data quality management, these platforms were reliant on the performance of the surveillance systems in different countries and related data sources. Data might be collected using different approaches with diverse rules for selection and inclusion, and their precision and representativeness is highly dependent on national resources and existing infrastructures. During the pandemic, the platform teams had limited capacity to directly engage with countries to address data quality improvements. However, by providing greater visibility of data generated by countries, the aggregation platforms may have played an indirect role to drive data quality improvement at the national level. Notwithstanding, for the teams to conduct data quality assessment and analytics, it was critical to understand the way data are collected by each surveillance system; unfortunately, the ‘metadata’ about surveillance system attributes, such as case definitions, or clinical and behavioural data, are not always easily available. This is why consolidating large volumes of data from disparate sources can facilitate the contextualisation of information and provide actionable insights to the public health workforce and policymakers. Although this was not discussed at length during the meeting, all platform teams are currently pushing towards a more efficient and precise data integration.

## Continuous adaptation and refinement

The course of the COVID-19 pandemic was shaped by the emergence of different SARS-CoV-2 variants, as well as the way epidemic control was managed in each country using public health and social measures, e.g. mask wearing, school closures [12], travel bans, and medical countermeasures such as vaccines, diagnostics and therapeutics [13]. The dynamic nature of the pandemic required an adaptive approach that enabled the different platforms to continuously refine their processes and analytic approaches. For example, with the Omicron variant of SARS-CoV-2 becoming dominant in most countries and vaccine coverage rising, metrics about test positivity became a weaker indication of epidemic trends; instead, hospitalisation rates and ICU admissions became more important to track the disease dynamics. The need to continually refine data interpretation was underscored by OWID, which frequently reviewed the metrics on their platform throughout the entire data pipeline from data aggregation, curation, and validation to visualisation and communication.

The dynamic nature of the pandemic is also a reminder that highly granular data are needed to understand if key epidemiological parameters are changing. Global.Health develops tools for researchers to combine external datasets, including genomic, clinical and policy data, with line-listed data for richer insights, analysis and actionable visualisations. The Global Initiative on Sharing All Influenza Data (GISAID, for genomic data) and the International Severe Acute Respiratory and Emerging Infection Consortium (ISARIC, for clinical data) are examples of data sources that can be integrated by Global.Health. Indeed, Global.Health was able to provide support to other projects with data integration, such as the collaboration with ISARIC to integrate clinical severity data on SARS-CoV-2 Omicron BA.1 to BA.5 with their line-listed datasets, and hence with a global data system [14].

## Providing transparency and building trust through open access

All platforms based their approaches on transparency, implementing open-source and open-access methods to create credibility and trust. Johns Hopkins University and OWID published their complete datasets as well as the source of each data point on their GitHub repositories [6,8]. Johns Hopkins University has consistently worked with teams of data modellers to create, organise and improve their extensive documentation of their GitHub repository with information on issues relevant to data modelling. Our World In Data publishes the code behind their analyses, stressing that although a small minority of users seek access to the code, their open-code policy has a high value as it creates clarity and trust around their decision-making processes. Similarly, Global.Health provides open access to their highly granular and de-identified line-listed datasets and visualisations through customisable downloads as well as application programming interfaces (APIs), making it possible for anyone to access and import the data into different platforms.

All three teams stressed that these approaches were enabling factors in attracting external contributions and volunteers that, in turn, were crucial to ensuring the reliability of their data and their success. Through GitHub repositories, via email and social media, volunteers communicated and collaborated directly with these platforms, not only by helping to improve data quality but also by building and curating the datasets to accurately inform decision- making. For OWID, for instance, crowdsourcing through volunteers was particularly crucial during the first 6 months of the establishment of the global vaccination dataset. Likewise, Global.Health incorporated an open-access curation tool into their data system enabling external contributors to digitise and standardise anonymised case data [15].

## Data privacy and security

The open-access nature of these platforms is a large part of their appeal and, as such, makes them an important contribution to the next generation of public health intelligence platforms. However, creating open-access public health information platforms must be balanced by data privacy and security concerns. Even though all three platforms featured during the Forum use data from publicly available sources, robust procedures to ensure data privacy are needed because not all public domain sources may adhere to good data privacy standards. This means understanding where the data are coming from, how they are used, what they are used for, and who is responsible for managing and maintaining it. Considering security, privacy, governance and ownership of the data throughout each step of curation, analysis and visualisation, as well as following regulations and laws, is paramount.

In the case of Global.Health, data privacy protection required additional care because, unlike platforms that work with aggregated data, this platform provides detailed line-listed datasets. The initiative worked with international legal experts to conduct a data protection impact assessment and ensure compliance with national and regional data governance and privacy laws, which led them to migrate their cloud storage to European Union/European Economic Area (EU/EEA) jurisdiction. Furthermore, Global.Health has developed strategies to implement data protection efforts in close cooperation with several partnering institutions. The initiative sought to work with existing platforms as well as newly created local instances of the Global.Health platform to decentralise data storage. The decentralisation of the data storage aimed to ensure that the private and public users, e.g. Ministries of Health, maintain ownership of their data. All initiatives must also ensure data security to protect the integrity and continued functionality of the platforms. To effectively manage those challenges, new solutions are being developed. Data mash and federated learning have the potential to revolutionise global collaboration by allowing for the distributed analysis of data without the need to share the data itself. Considering that the public health data can be used to make decisions that have major impact on society, such as travel restrictions, it is important that these data platforms are reliable and accountable, as it is often the national public health authorities who are asked to comment on discrepancies or errors.

## Enhancing users’ engagement with the data

Each of the three teams worked alongside communications experts and designers to create visualisations of the data for users. Furthermore, they provided information on the particularities of the data, their methodologies and the characteristics of the data sources. The careful compilation of the ‘metadata’ provided an important added value to these platforms, as it enabled users to assess the quality of the data and to take that into account when interpreting analytic output.

Because of the far-reaching societal impacts of the COVID-19 pandemic, the communication component of these platforms became increasingly important and extended to users beyond government agencies and public health specialists. Taking this need for accessible and reliable scientific information into account, contributors of these platforms published non-technical articles on subjects related to the pandemic targeted at the general public. Examples of these user-centric efforts are the Pandemic Data Initiative (PDI) [16] developed by members of the Coronavirus Resource Center at JHU and the articles released by OWID. In addition to providing easily accessible analyses of their information, an important feature of all platforms was that they facilitated data downloads for people who wanted to conduct their own analysis.

## Promoting data sharing by governments

A persistent challenge for all three initiatives has been the fragmented and inconsistent data-sharing practices of national and local government agencies. In many countries, there has been a lack of COVID-19-dedicated web-based, accessible datasets. In some lower-income countries, governments have limited resources and capacities to establish a sufficient data-sharing infrastructure, such as lack of secure internet connection, IT gadgets or staff dedicated to those activities. Some government agencies used social media channels for sharing data with the public. Many countries also used external service providers to create dashboards without developing an in-house capacity for their management and maintenance. In other examples, government agencies published data visualisations without access to the underlying datasets.

Additional challenges for data aggregation included the lack of ‘metadata’ that suitably documented that data structures and the use of a wide variety of different metrics, sometimes without clear definitions. This limited opportunities to understand the data produced and shared by different government agencies and from different countries. One example of this was the inconsistency of reporting test data between countries [17], as different government agencies reported on differing test types and/or counting methods, resulting in incomparable case count data.

During the pandemic, there was some discussion on how local and national governments could share data using standardised protocols that facilitate data aggregation. Johns Hopkins University experienced that many public health agencies were willing to adapt their data-sharing practices upon request and even proactively asked for guidance on data-sharing standards to facilitate better analysis. Based on these experiences, there is a need for robust common protocols that define the frequency, scope, level of disaggregation and metadata for effective data sharing during epidemics and pandemics. The data would need to meet the principles of findability, accessibility, interoperability and reusability (FAIR). The FAIR principles emphasise machine-actionability, i.e. the capacity of computational systems to find, access, interoperate and reuse data with minimal or no human intervention, because humans increasingly rely on computational support to deal with data as a result of the increase in volume, complexity, and creation speed of data. There is also the need for better tools to share and compile aggregated data that leverage opportunities in data science to automate much of the workflow, which may even lessen the burden for regions and countries when fulfilling their International Health Regulations (IHR) obligation to report data.

## Ways forward

It is clear that non-government actors can play an important role in bringing innovation and creativity to the work of global public health surveillance. Because the public health sector is often under-funded and has limited access to specialists in data science and new technologies, the engagement by academic and philanthropic entities should be encouraged. Additional opportunities include the use of crowdsourcing approaches to maximise the application of new and cutting-edge approaches to working with public health data. It is also important to have the contribution of different voices and perspectives from outside the government sector to generate a deeper reflection about the meaning of public health data.

The work of aggregator initiatives highlights the need for government agencies to improve data-sharing protocols that include clear privacy protection standards and comprehensive metadata on the data structure and sources. It is necessary for entities such as WHO to facilitate the establishment of global data-sharing standards and procedures for public health emergencies. Moreover, there is an urgent need at the country level to help public health agencies adopt the already available tools for data management or develop new ones if the existing tools do not fit their needs.

It is likely that the work of non-governmental aggregator initiatives during the pandemic also increased engagement with a wider range of stakeholders. This improved engagement likely had additional benefits, such as improving public health literacy overall and increasing engagement with members of the public; community engagement is one of the most important factors that determines the success of outbreak control efforts. It is also important to consider that – in a world where data flow ever faster and wider – our communities have expectations of more openness and transparency of data collected by government entities [18].

The provision of open-access aggregated datasets by non-governmental initiatives during the pandemic has been transformative for global public health surveillance. Further engagement with non-governmental surveillance efforts can accelerate the necessary improvements needed in public health surveillance overall. Increasing the diversity of public health surveillance initiatives beyond the government sector comes with several benefits: accelerated adoption of data science methodologies, engagement of additional highly skilled professionals, greater transparency and accountability for government agencies, and new opportunities to engage with members of our society.

## Conclusions

To be prepared for the next pandemic and ensure sustainability of public health intelligence efforts in the meantime, countries must work diligently to create concepts that will justify sustained budgetary support and resources. Given the continued risk of outbreaks or climate-sensitive disasters, it is essential to continue using the data systems and the surveillance initiatives developed during the pandemic to monitor and respond to future outbreaks. This requires sufficient funding, particularly to maintain the right number and type of personnel in the workforce. Leveraging lessons from the pandemic and resources from other global funding streams, such as The Pandemic Fund [19] and Climate Change Fund [20], could be instrumental in this task. Also, investing in data collection from traditional sources and non-traditional sources could be a key component to a sustainable model. Additionally, robust networks must be built with multiple avenues of support, as well as incentives to encourage collaboration and prevent destructive competition.
